# Effectiveness of lotilaner on furuncular myiasis in dogs naturally infested with *Dermatobia hominis* (Diptera: Cuterebridae)

**DOI:** 10.1590/S1984-29612024054.

**Published:** 2024-09-16

**Authors:** Rafaella Tortoriello, Luisa Xavier Christ, Victoria Caroline de Almeida Marques, Bruna Sampaio Martins, Julio Israel Fernandes

**Affiliations:** 1 Programa de Pós-graduação em Ciências Veterinárias – PPGCV, Instituto de Veterinária, Universidade Federal Rural do Rio de Janeiro – UFRRJ, Seropédica, RJ, Brasil; 2 Instituto de Veterinária, Universidade Federal Rural do Rio de Janeiro – UFRRJ, Seropédica, RJ, Brasil; 3 Programa de Pós-graduação em Medicina Veterinária – PPGMV, Instituto de Veterinária, Universidade Federal Rural do Rio de Janeiro – UFRRJ, Seropédica, RJ, Brasil; 4 Departamento de Medicina e Cirurgia Veterinária, Instituto de Veterinária, Universidade Federal Rural do Rio de Janeiro – UFRRJ, Seropédica, RJ, Brasil

**Keywords:** Dermatobia hominis, lotilaner, treatment, isoxazolines, Dermatobia hominis, lotilaner, tratamento, isoxazolinas

## Abstract

An evaluation was made of the larvicidal efficacy of lotilaner (Credeli®) in the treatment of dogs naturally infested with *Dermatobia hominis* larvae. A total of 12 dogs presenting at least three live *D. hominis* larvae were medicated. The animals were medicated orally with a single dose of no less than 20 mg/kg lotilaner. After drug administration, the animals remained at their homes, and observations were made to verify the larvicidal effect 6 hours after treatment. Live larvae were considered any parasite that exhibited motility after removal. For each animal was using the formula: 100 x [(total of live larvae before treatment − total live larvae after treatment) /total of live larvae before treatment] as criteria for evaluating lotilaner efficacy. A total of 98 larvae were counted in 12 dogs, with an average of 8.1 larvae per animal. The effectiveness of lotilaner was 80.6%. Nineteen larvae were found alive, albeit presenting hypomobility and lethargic behavior. However, note that the evaluation was performed just six hours after administration of the drug. Lotilaner administered orally in a single dose of 20 mg/kg showed 80.6% efficacy six hours after treating dogs naturally infested with *D. hominis*.

## Resumo

Foi avaliada a eficácia larvicida do lotilaner (Credeli®) no tratamento de cães naturalmente infestados com larvas de *Dermatobia hominis*. Foram medicados 12 cães, apresentando pelo menos três larvas vivas de *D. hominis*. Os animais foram medicados por via oral com dose única não inferior a 20 mg/kg de lotilaner. Após a administração desse medicamento, os animais permaneceram em seus domicílios, e foram feitas observações para verificar o efeito larvicida 6 horas após o tratamento. Larvas vivas foram consideradas quaisquer parasitas que exibissem motilidade após a remoção. Para cada animal foi utilizada a fórmula: 100 x [(total de larvas vivas antes do tratamento - total de larvas vivas após o tratamento) /total de larvas vivas antes do tratamento] como critério para avaliar a eficácia do lotilaner. Foram contadas 98 larvas em 12 cães, com média de 8,1 larvas por animal. A eficácia do lotilaner foi de 80,6%. Dezenove larvas foram encontradas vivas, porém apresentando hipomobilidade e comportamento letárgico. No entanto, vale ressaltar que a avaliação foi realizada apenas 6 horas após a administração do medicamento. Lotilaner, administrado por via oral em dose única de 20 mg/kg, apresentou eficácia de 80,6%, seis horas após o tratamento de cães naturalmente infestados por *D. hominis*.

## Introduction

*Dermatobia hominis* (Linnaeus, Jr, 1781) (Diptera: Cuterebridae), commonly known as the human botfly, is an obligate parasite whose larvae parasitizes a wide range of vertebrate hosts, including domestic or wild animals (Verocai et al., 2010). This parasitic species primarily causes cutaneous myiasis. The animals most susceptible to this parasite are cattle, while dogs are the most commonly parasitized companion animals, although feline cases have also been described (Verocai et al., 2010; Scholl et al., 2019), and a few human cases have been reported (Roncalli & Benitez Usher, 1988; McGraw & Turiansky, 2008).

*D. hominis* commonly occurs in wooded areas, where the average temperature is 20°C and relative humidity ranges from 85% to 95% (Roncalli & Benitez Usher, 1988). This dipteran can be found in Latin America from southern Mexico to northern Argentina (McGraw & Turiansky, 2008; Rojas et al., 2011) and is widely present in Brazil, where many animals living in infested regions become parasitized.

First instar larvae penetrate intact or damaged skin. Reddish nodules form with a central hole from which the human botfly’s back end occasionally protrudes. Larval movements cause restlessness and irritation, and heat and scratching can lead to nodule ulceration and bacterial invasions, as well as secondary myiasis and abscesses (Rojas et al., 2011).

Diagnosis is based on the visual detection of larva and fistula and confirmed via skin compression revealing larvae (McGraw & Turiansky, 2008). Animals may be parasitized with just one larva or, in more severe cases, with several nodules containing larvae (McGraw & Turiansky, 2008).

Recent publications provide guidelines for the mechanical control of companion animals, which causes intense discomfort, sometimes requiring sedation in heavily infested animals (Cramer-Ribeiro et al., 2002; Moya Borja, 2003; Neves et al., 2015; Junquera et al., 2019), although several curative and preventive protocols are available for the treatment of large animals. In addition, there are chemical products and medications that serve to control and eliminate larvae, such as macrocyclic lactones (Neves et al., 2015), benzoylureas (Junquera et al., 2019), organophosphates (Rojas et al., 2011), and isoxazolines (Andriotti et al., 2021; Campos et al., 2021).

Lotilaner is a potent inhibitor of the neurotransmitter gamma–aminobutyric acid and glutamate receptors, acting on the neuromuscular junction in insects, resulting in uncontrolled neuromuscular activity, which leads to the rapid death of insects and mites (Kuntz & Kammanadiminti, 2017). This molecule has the fastest peak of action, and its bioavailability is enhanced by administering it after feeding (Kuntz & Kammanadiminti, 2017; Cavalleri et al., 2017; Toutain et al., 2017; Zhou et al., 2022).

Recent studies have used isoxazolines for myiasis treatment with excellent results (Oliveira et al., 2019; Han & Yasmin, 2020; Andriotti et al., 2021; Campos et al., 2021; Vale et al., 2023). Sarolaner and topic fluralaner demonstrated 100% effectiveness against *D. hominis* (Andriotti et al., 2021; Campos et al., 2021). Sarolaner is also effective against *Cochliomyia hominivorax* (Oliveira et al., 2019), while lotilaner is effective against *Chrysomya bezziana* and *Cochliomyia hominivorax* (Han & Yasmin, 2020; Vale et al., 2023). However, reports on the use of lotilaner in dogs infested with *D. hominis* larvae were not found in the literature.

This study aimed to evaluate the larvicidal effectiveness of lotilaner in the treatment of dogs naturally infested with *D. hominis*.

## Methods

A total of 12 male and female dogs of mixed breed, between 1 and 10 years old, weighing from 10 to 20 kg, were selected after obtaining authorization from their owners. The inclusion criteria were as follows: none of the animals could have undergone any type of treatment against ticks, fleas, acaricides, and/or larvicides in the last 90 days preceding the study, and they had to present at least three myiases.

The animals underwent a physical examination to assess the general condition, location, and quantification of larvae.

Following the methodology of Andriotti et al. (2021), Campos et al. (2021) and Scholl et al. (2019) the diagnosis was clinically achieved after performing trichotomy and cleaning of the lesion by observing skin nodules and detecting larval. Any larva after exerting digital compression that showed motility during the examination was considered alive. Larvae were later identified and morphologically classified.

The animals were orally medicated with a single dose of at least 20 mg/kg of lotilaner (Credeli® - Elanco), following the instructions on the package insert for other ectoparasite treatments.

All the animals were examined 6 h after treatment. In this study, the animals were the controls. The following formula was used: 100 x [(total of live larvae before treatment − total live larvae after treatment) /total of live larvae before treatment] as criteria for evaluating the larvicidal efficacy of lotilaner.

## Results

No adverse effects were reported in the animals that participated in this study after the administration of lotilaner. The lesions on these dogs consisted of rigid, inflamed, erythematous epidermis with a nodular and papular appearance and the presence of a central hole with or without serosanguineous discharge. Table 1 describes the location and number of larvae in the 12 animals included in the study. Of the 12 affected dogs, 50% (6) were male and 50% (6) were female. The average age was 3 years, with a weight between 10 and 20 kg. All the animals had short fur and were of mixed breed.

**Table 1:** The total number of dogs evaluated the respective totality of *Dermatobia hominis* live larvae in each animal, and the location of their lesions in two periods distinct (T0 and T+6h).

Animals	Weight	Dose (mg/Kg)	Sex	Ears	Head	Side	Tail	Limbs	Back	Belly	Total T0 T+6h	
1	17	26.4	F	1	2	1					4	
2	16	28.1	M			3			3		6	
3	11	38.4	F					4			4	
4	11	40.1	F					2	1		3	
5	19	23.7	M					1	5		6	4
6	15	30	M					7	8		15	
7	12	36.8	F				1	1	7		9	
8	14	31	M					3	1		4	
9	18	24.6	M			1		2	1		4	
10	20	22.5	F					6			6	2
11	17	25.7	M		1	12	2	5	10		30	13
12	18	24.5	F			1	5			1	7	
Total	11	29.5		1	1	18	8	31	36	1	98	19

M: male; F: female; T0: Drug administration; T+6h: Time for larvae removal.

A total of 98 *D. hominis* larvae were collected. One of the dogs had the lowest parasite load within the inclusion criteria, i.e., three larvae, while four others had four larvae each. The average parasite load was 8.1 larvae per dog, with one animal having 30 larvae.

The location of larvae varied among the animals. However, the highest concentration of larvae was found in the animal’s dorsal region, although a very similar number was also found in the limbs.

Six hours after the administration of lotilaner, digital compression was performed, resulting in the removal of all the larvae, which were evaluated for motility. Treatment efficacy was 80.6%, with the death of 79 larvae and the survival of 19 larvae six hours after administration of the drug. However, hypomobility was evident and recorded among the 19 surviving larvae.

## Discussion

Treatment via mechanical removal through digital compression is still a routine procedure, but it usually causes discomfort and eventually moderate to severe pain in the animal (Roncalli & Benitez Usher, 1988). This study aimed to verify the larvicidal efficacy of lotilaner against *D. hominis*.

Our evaluation time was chosen because, according to Cavalleri et al. (2017), lotilaner demonstrated the fastest flea-killing speed among isoxazolines, showing 98% efficacy against fleas at 6 hours post-treatment. And following the methodology of Andriotti et al. (2021) and Campos et al. (2021), motility was evaluated after the larvae were removed by digital compression and placed on a flat, smooth surface to verify the presence of movement.

Lotilaner provides one of the highest levels of bioavailability, reaching its peak in its plasma concentration more quickly than do other isoxazolines (Kuntz & Kammanadiminti, 2017; Cavalleri et al., 2017; Toutain et al., 2017). In the present study, lotilaner was chosen due to its rapid action peak, and showed 80.6% efficacy 6 hours after administration of the drug. When compared with a study in which lotilaner was used in cats against *C. bezziana*, and to two other reports, one in which sarolaner was used in dogs and another topic fluralaner in cats, the efficacy achieved in our study was lower. This can probably be attributed to the methodology we used, since the drug’s efficacy was verified after just 6 hours, thus differing from the other three reports in which the drugs were evaluated in a period ranging from 24 to 48 h after their administration (Han & Yasmin, 2020; Andriotti et al., 2021; Campos et al., 2021).

The larvae were removed after 6 hours to ensure that they were dead or with little motility, thereby avoiding data misinterpretation. In addition, it is worth noting that the larvae were removed easily, even the live ones, and caused less discomfort to the animals, as observed in another study (Andriotti et al., 2021). Therefore, we believe that if our assessment of the efficacy of lotilaner had been carried out within 24 hours, a methodology similar to that used in other studies (Andriotti et al., 2021), the drug would have exhibited a similar efficacy.

However, it is important to highlight that the evaluation carried out 6 hours after administration does not allow us to deduce what would happen to the 19 larvae found alive if they had not been removed. But it is likely that they would die within a few hours, but they could also remain alive and complete their cycle.

The location of lesions in this study was similar to those reported in another study (Cramer-Ribeiro et al., 2002), which described the dorsal region as being the most affected (36.7%), followed by the limbs. However, the same author (Cramer-Ribeiro et al., 2002) demonstrated a higher prevalence of myiasis in males, while preference for a specific sex was not observed in this study. However, the findings in this work differed from those of previous studies, which found that the most prevalent site was bilateral chest areas (Andriotti et al., 2021).

All the dogs in this study had short fur, a characteristic that may have influenced the high incidence of parasitized animals. As reported by other authors (Cramer-Ribeiro et al., 2002; Andriotti et al., 2021), the type of coat may be a predisposing factor for parasitism. However, a single animal presented a greater parasitism (n 30) when compared to others, it is believed that occurred as it slept with farm animals in a stable.

Finally, 30 days after administration of the drug, the owners of the animals that participated in the study were contacted and reported no new reinfestations. The same finding was reported in another study, which also used sarolaner, an isoxazoline (Andriotti et al., 2021).

## Conclusion

A single dose of 20 mg/kg of lotilaner administered orally after six hours demonstrated 80.6% efficacy in treating myiasis in dogs naturally infested with *Dermatobia hominis*.
